# Complete Genome Sequence of a 2019 Novel Coronavirus (SARS-CoV-2) Strain Isolated in Nepal

**DOI:** 10.1128/MRA.00169-20

**Published:** 2020-03-12

**Authors:** Ranjit Sah, Alfonso J. Rodriguez-Morales, Runa Jha, Daniel K. W. Chu, Haogao Gu, Malik Peiris, Anup Bastola, Bibek Kumar Lal, Hemant Chanda Ojha, Ali A. Rabaan, Lysien I. Zambrano, Anthony Costello, Kouichi Morita, Basu Dev Pandey, Leo L. M. Poon

**Affiliations:** a National Public Health Laboratory, Kathmandu, Nepal; b Public Health and Infection Research Group, Faculty of Health Sciences, Universidad Tecnologica de Pereira, Pereira, Risaralda, Colombia; c Grupo de Investigación Biomedicina, Faculty of Medicine, Fundación Universitaria Autónoma de las Américas, Pereira, Risaralda, Colombia; d School of Public Health, LKS Faculty of Medicine, The University of Hong Kong, Hong Kong Special Administrative Region, China; e Sukraraj Tropical and Infectious Disease Hospital, Kathmandu, Nepal; f Epidemiology and Disease Control Division, Government of Nepal, Kathmandu, Nepal; g Johns Hopkins Aramco Healthcare, Dhahran, Saudi Arabia; h Departments of Physiological and Morphological Sciences, School of Medical Sciences, Universidad Nacional Autónoma de Honduras (UNAH), Tegucigalpa, Honduras; i Global Health and Sustainable Development, University College London, London, United Kingdom; j Department of Virology, Institute of Tropical Medicine, Nagasaki University, Nagasaki, Japan; DOE Joint Genome Institute

## Abstract

A complete genome sequence was obtained for a severe acute respiratory syndrome coronavirus 2 (SARS-CoV-2) strain isolated from an oropharyngeal swab specimen of a Nepalese patient with coronavirus disease 2019 (COVID-19), who had returned to Nepal after traveling to Wuhan, China.

## ANNOUNCEMENT

Severe acute respiratory syndrome coronavirus 2 (SARS-CoV-2), family *Coronaviridae*, genus *Betacoronavirus*, is spreading widely in China, causing coronavirus disease 2019 (COVID-19) (1), and is also affecting other Asian and non-Asian countries (2, 3). Imported cases have been reported in countries such as Japan, Singapore, Hong Kong, Thailand, and Nepal, among others (4). We report here the complete genome sequence of SARS-CoV-2 from a Nepalese patient; the infection was acquired in Wuhan, China, and imported to Nepal.

The isolate (BetaCoV/Nepal/61/2020) is from the oropharyngeal swab specimen of a 32-year-old man, a Nepalese student at Wuhan University of Technology in Wuhan, China, with no history of comorbidities, who returned to Nepal presenting with cough, mild fever, and throat congestion, suggesting COVID-19 (4). An oropharyngeal swab specimen was collected at the National Influenza Centre, National Public Health Laboratory in Kathmandu, Nepal, and submitted to the WHO laboratory at the University of Hong Kong, Hong Kong Special Administrative Region, China, where it was confirmed and sequenced.

The specimen tested positive for SARS-CoV-2 by real-time reverse transcriptase PCR (rRT-PCR) developed in the University of Hong Kong (5). Sequencing was done using the Illumina MiSeq system with the Burrows-Wheeler Aligner MEM algorithm (BWA-MEM) 0.7.5a-r405 assembly method. The full genome was amplified directly from the RNA extract from the original specimen using gene-specific primers for open reading frame 1b (ORF1b) and N (Table 1) to produce overlapping PCR products covering the full genome (5). The expected amplicon sizes of the ORF1b and N gene assays are 132 bp and 110 bp, respectively (5). The raw reads were first cleaned by trimming low-quality bases with Trimmomatic 0.36 (-phred33, LEADING:20, TRAILING:20, SLIDINGWINDOW:4:20, MINLEN:40). The new genome sequence was obtained by first mapping reads to a reference SARS-CoV-2 genome using BWA-MEM 0.7.5a-r405 with default parameters to generate the consensus sequence. In addition, the assembly produced by MEGAHIT 1.2.9 (*de novo* assembly), using default parameters, was used to cross-validate with the reference-based method as an internal control. The two results were consistent, and our final sequence is based on the reference-based method. The reference sequence we used was from the Global Initiative on Sharing All Influenza Database (GISAID; strain identifier EPI_ISL_405839). The reads mapped to the reference sequence were then curated in a pileup alignment file to obtain the consensus sequence (minimum coverage threshold, 10). FastQC 0.11.8 was used to assess the sequence quality before trimming and after alignment to prevent potential errors. There were 5,246,584 paired-end sequences in the raw data. A total of 9,891,431 records were included in the reference-based alignment after trimming, and 9,887,093 (99.96%) of them were mapped to the SARS-CoV-2 reference genome.

**TABLE 1:** Gene-specific primer and probe sequences used

Gene	Primer^*a*^
ORF1b	
Forward	5′-TGGGGYTTTACRGGTAACCT-3′
Reverse	5′-AACRCGCTTAACAAAGCACTC-3′
Probe	5′-TAGTTGTGATGCWATCATGACTAG-3′^*b*^
N	
Forward	5′-TAATCAGACAAGGAACTGATTA-3′
Reverse	5′-CGAAGGTGTGACTTCCATG-3′
Probe	5′-GCAAATTGTGCAATTTGCGG-3′^*b*^

a Y is C or T; R is A or G; W is A or T.

b In 5′-6-carboxyfluorescein/ZEN internal quencher/3′-Iowa Black fluorescent quencher format.

We generated a consensus sequence of 29,811 bp with no gap and high average coverage (>77,000×). Primer binding sites at the 5′ and 3′ ends were removed, resulting in this genome being 59 nucleotides (nt) shorter than a reference genome in GenBank (accession number NC_045512), excluding the poly(A) tail of the genome.

For phylogenetic analyses, SARS-CoV-2 full-genome sequences were aligned with CLUSTAL W (6) using MEGA 10.0.5. (7). The new SARS-CoV-2 sequence was compared to existing genomes using online NCBI BLAST (https://blast.ncbi.nlm.nih.gov/Blast.cgi).

Full-genome comparison of the isolate revealed >99.99% identity with two previously sequenced genomes available at GenBank (MN988668 and NC_045512) for SARS-CoV-2 from Wuhan, China, and >99.9% with seven additional sequences (MN938384.1, MN975262.1, MN985325.1, MN988713.1, MN994467.1, MN994468.1, and MN997409.1). The final genome of sequenced SARS-CoV-2 consists of a single, positive-stranded RNA that is 29,811 nucleotides long, broken down as follows: 8,903 (29.86%) adenosines, 5,482 (18.39%) cytosines, 5,852 (19.63%) guanines, and 9,574 (32.12%) thymines.

The sequence of BetaCoV/Nepal/61/2020 from coordinates 1 to 29811 is identical to the sequence of isolate 2019-nCoV WHU01 (GenBank accession number MN988668) from 15 to 29825 (29810/29811), except at site 24019, with a substitution of a C, from 2019-nCoV WHU01, for T. The sequence of BetaCoV/Nepal/61/2020 from coordinates 1 to 29811 is identical to the sequence of isolate Wuhan-Hu-1 (GenBank accession number NC_045512) from 16 to 29826 (29810/29811), except at site 24019, with the same substitution of a C from isolate Wuhan-Hu-1 for T.

The C24019T mutation corresponds to C24034T if we use the sequence located under GISAID strain identifier EPI_ISL_405839 as a reference. This was a silent mutation at the spike gene (codon AAC to AAT). Based on the reference sequence, the following five mutations were also identified: T8782C (in ORF1a, codons AGT to AGC, silent mutation), T9561C (in ORF1a, codons TTA to TCA, nonsilent mutation), C15607T (in ORF1b, codons CTA to TTA, silent mutation), C28144T (in ORF8b, codons TCA to TTA, nonsilent mutation), and T29095C (in nucleocapsid, codons TTT to TTC, silent mutation).

Additional epidemiological and clinical features of this case of COVID-19 were reported in reference 4.

### Data availability.

This sequence has been deposited in GenBank under the accession number MT072688 and at the GISAID EpiCoV newly emerging coronavirus SARS-CoV-2 platform under identifier EPI_ISL_410301. The accession numbers for the Illumina MiSeq sequence raw reads in the NCBI Sequence Read Archive (SRA) are PRJNA608651 (BioProject), SRP250653 (SRA), SAMN14180202 (BioSample, BetaCoV/Nepal/61/2020), SRX7798477 (SRA; GISAID EPI_ISL_410301), and SRR11177792 (run, WHV-Nepal-61-TW_1.fastq.gz).
