# Expression of Concern for Sah et al., “Complete Genome Sequence of a 2019 Novel Coronavirus (SARS-CoV-2) Strain Isolated in Nepal”

**DOI:** 10.1128/MRA.01466-20

**Published:** 2021-01-21

**Authors:** American Society for Microbiology

**Affiliations:** Washington, DC, USA

## EXPRESSION OF CONCERN

The American Society for Microbiology (ASM) and *Microbiology Resource Announcements* are issuing this Expression of Concern regarding the following publication:

Sah R, Rodriguez-Morales AJ, Jha R, Chu DKW, Gu H, Peiris M, Bastola A, Lal BK, Ojha HC, Rabaan AA, Zambrano LI, Costello A, Morita K, Pandey BD, Poon LLM. 2021. Complete genome sequence of a 2019 novel coronavirus (SARS-CoV-2) strain isolated in Nepal. Microbiol Resour Announc 9:e00169-20. https://doi.org/10.1128/MRA.00169-20.

*Microbiology Resource Announcements* was notified by the Nepal Health Research Council (NHRC) that the authors did not have ethics approval from the NHRC, which the NHRC alleges to be required for such research in Nepal. The authors provided documentation to ASM that informed, written consent was taken from the patient and that the research was performed in accordance with the Declaration of Helsinki, as revised in 2013. The authors informed the ASM that they filed and registered a petition in the Supreme Court of Nepal to challenge the NHRC’s claim that NHRC approval is needed for such type of publication. This Expression of Concern is issued pending the final court decision and will be updated accordingly thereafter.

